# Association of immune checkpoint inhibitors with muscle mass and density in patients with melanoma

**DOI:** 10.1093/oncolo/oyag029

**Published:** 2026-02-12

**Authors:** Susan Ziolkowski, Bryn E Matheson, Steven K Boyd, Matthias Walle, Jasmine Gill, John Walker, Thomas Salopek, Joshua F Baker, Ates Fettahoglu, Carrie Ye

**Affiliations:** Department of Medicine, Stanford University, Palo Alto, CA 94305, United States; McCaig Institute for Bone and Joint Health, Cumming School of Medicine, University of Calgary, Calgary, AB T6G 2R3, Canada; McCaig Institute for Bone and Joint Health, Cumming School of Medicine, University of Calgary, Calgary, AB T6G 2R3, Canada; McCaig Institute for Bone and Joint Health, Cumming School of Medicine, University of Calgary, Calgary, AB T6G 2R3, Canada; Department of Medicine, University of Alberta, Edmonton, AB T2N 4Z6, Canada; Department of Medicine, University of Alberta, Edmonton, AB T2N 4Z6, Canada; Department of Medicine, University of Alberta, Edmonton, AB T2N 4Z6, Canada; Corporal Michael J. Crescenz VA Medical Center, Philadelphia, PA 19104, United States; Perelman School of Medicine at the University of Pennsylvania, Philadelphia, PA 19104, United States; Department of Epidemiology and Biostatistics, University of Pennsylvania, Philadelphia, PA 19104, United States; Department of Radiology, Stanford University, Palo Alto, CA 94305, United States; Department of Medicine, University of Alberta, Edmonton, AB T2N 4Z6, Canada; Arthritis Research Canada, Vancouver, V5Y 3P2, Canada

**Keywords:** immunotherapy, kidney disease, melanoma, immune-related adverse events

## Abstract

**Background:**

The association between skeletal muscle wasting and immune checkpoint inhibitor (ICI) use for cancer and whether pre–treatment kidney function modifies this relationship is unknown.

**Materials and Methods:**

Single center retrospective cohort study of patients with non–metastatic melanoma treated with or without ICIs, with a pre–treatment CT or PET-CT scan and follow-up scan within 1 year (±3 months). Paired *t*-tests examined the change in abdominal skeletal muscle index (SMI, cm^2^/height in meters) and psoas muscle density (PMD, Hounsfield units) at L3. Independent *t*-tests compared differences in the mean change between the ICI and non–ICI groups. Multivariable linear regression models assessed whether the change in SMI and PMD was different between groups and whether baseline estimated glomerular filtration rate (eGFR, mL/min/1.73 m^2^) modified this association.

**Results:**

Thirty-six patients treated with ICIs and 41 not treated with ICIs met inclusion criteria. The non–ICI group was older (mean age ± SD = 67.0 ± 12.9 years) and more commonly male (78%) compared to the ICI group (58.9 ± 14.9 years; 50% male). The mean change in SMI was not significantly different between groups (*P* = 0.38). The mean change in PMD in the ICI group was −5.93 (95% confidence interval [CI] −9.58 to −2.28), which was significantly greater than the non–ICI group (−1.18, 95% CI −3.51 to 1.15), after adjusting for baseline variables (*P* = 0.002). Findings were not affected by baseline eGFR.

**Conclusion:**

Patients treated with ICIs had a significantly higher decline in PMD as compared to patients not treated with ICIs, which was not affected by baseline eGFR.

Implications for Clinical Practice In this single-center retrospective cohort of patients with non–metastatic melanoma, skeletal muscle changes were compared between patients treated with or without immune checkpoint inhibitors (ICIs). Changes in abdominal skeletal muscle index (SMI) and psoas muscle density (PMD) were assessed on CT or PET-CT imaging at baseline and at 1 year. Changes in SMI did not differ between groups; however the ICI group experienced a significantly greater decline in PMD compared with the non–ICI group. Baseline kidney function did not modify this association. Clinicians should be aware of a potential decline in psoas muscle quality in patients treated with ICIs as this outcome has been associated with poor balance, declines in physical function, increased hospitalizations, and decreased lifespan.

## Introduction

Immune checkpoint inhibitors (ICIs) have revolutionized cancer treatment by exerting anti-tumor effects on various types and stages of cancer. These monoclonal antibodies target immune checkpoints, including programmed death 1 (PD1) and cytotoxic T-lymphocyte–associated protein 4 (CTLA-4), to augment the immune response against tumor cells. Releasing the brakes on the immune system has consequences, however, in the form of immune-related adverse events (irAEs), which may affect any organ.

A potential side effect of ICI therapy is immune-related myositis, a condition causing muscle inflammation and weakness, which can further contribute to muscle mass loss.^1^ Muscle histopathology of patients with ICI myositis describes a unique signature of multifocal clusters of necrotic and regenerating fibers, differentiating ICI myopathy from other autoimmune myopathies.^2^ Transcriptomic analysis has uncovered interferon pathway activation and notable upregulation of the interleukin (IL)-6 pathway in affected muscle tissue.^3^ While the incidence of myositis is estimated at 0.38%,^4^ the occurrence of more indolent skeletal muscle wasting while on ICIs is unknown.

Among patients treated with ICIs, pre–treatment sarcopenia (ie, low muscle mass) is associated with poorer overall survival and progression-free survival in patients.^5^ Further, chronic kidney disease (CKD) is associated with sarcopenia^6^ and cancer-related mortality is significantly higher among patients with CKD.^7^ Therefore, determining whether pre–treatment kidney function affects the change in muscle mass and density while on ICI therapy will help determine safety of these drugs for patients with poor baseline kidney function prior to drug start.

The availability of abdominal computed tomography (CT) scans is nearly universal in the cancer population. Measurements of skeletal muscle mass can be extracted from abdominal CT via a machine learning assisted program to compute skeletal muscle index (SMI). SMI is a measure of skeletal muscle area (SMA) at the third lumbar spine vertebrae (L3) indexed to patient height and has been significantly associated with mortality in a retrospective cohort study of 16 575 patients at Stanford Health Care.^8^ CT-measured abdominal muscle density (PMD), which measures the average Hounsfield unit (HU) attenuation of the bilateral abdominal muscles, is a promising predictor of mortality in patients with various cancers,^9^^,^^10^ type 2 diabetes mellitus,^11^ and those on hemodialysis. Both low SMI and PMD have been associated with decreased overall and melanoma specific survival.^12^

The aim of this study was to utilize clinical CT scans from patients with melanoma to compare the impact of ICI therapy on SMI and PMD and assess the impact of baseline estimated glomerular filtration rate (eGFR) on these measurements. We hypothesize that patients treated with ICIs will have larger changes in SMI and PMD as compared to patients not treated with ICIs and that lower baseline eGFR accelerates this change.

## Methods

### Patient selection

We previously described our cohort selection^13^ as part of a retrospective cohort study involving melanoma patients treated at the Cross Cancer Institute or Kaye Edmonton Clinic in Alberta, Canada, between October 2016 and October 2021. The study included melanoma patients who received ICI therapy (referred to as the ICI group), with both a baseline CT or PET-CT scan and a follow-up scan within 1 year (±3 months). The ICI group was defined as patients with stage III melanoma who received at least 6 months of ICI therapy. The control group, referred to as non–ICI group, consisted of patients with stage II melanoma who had a baseline CT or PET-CT but did not receive any ICI therapy or other systemic cancer therapies prior to their follow-up scan. We excluded patients who were under 18, had bone metastasis at baseline or any time before the follow-up scan, had hardware, instrumentation, vertebral fractures, or other structural abnormalities in the lumbar spine, or had used any of the following medications prior to the follow-up scan: glucocorticoids (≥5 mg prednisone-equivalent for 3 months or more), androgen deprivation therapy (ADT), aromatase inhibitors, or osteoporosis medications.

### Image parameters

The clinical scans used in the CT analysis were acquired from a variety of manufacturers, CT scanner models and imaging protocols. Forty-six scans were taken on a GE Healthcare CT scanner, 42 on a Siemens CT scanner, 39 on a Canon Medical Systems scanner and 27 on a Philips scanner (Table S1—see online supplementary material). The make of the baseline scanner differed from follow-up scanner in 66.67% and 53.66% of the ICI and non–ICI group, respectively.

### Extraction of body composition measurements

**Figure 1.**
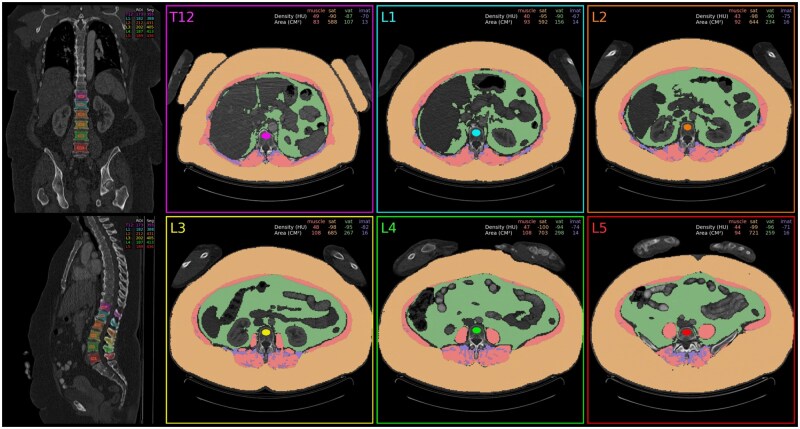
Segmentations used for quantitative skeletal muscle index analysis.

We utilized TotalSegmentator, an open-source package for rapid and automated body composition analysis of CT scans, to segment skeletal muscle, subcutaneous abdominal tissue, visceral adipose tissue and intermuscular adipose tissue (IMAT) (Figure 1).^14^ Total segmentator was developed using manual annotation of 1204 CT examinations that served as the ground truth for training and testing. The resulting masks were used to compute body composition measures for skeletal muscle and adipose tissue from CT scans. SMI was computed from SMA at the third lumbar vertebra (L3; cm^2^) divided by patient height (m), consistent with previous work.^8^ PMD was measured as the mean HU attenuation of the bilateral abdominal muscles calculated at the L3 level.

Low PMD was assessed using the following diagnostic cut-off values: (1) non sex-specific <38.5^15^; and (2) sex-specific cutoffs of <40.5 HU for men and <28.4 HU for women.^16^ e manually reviewed CT reports for mention of myositis or disease progression.

### Estimates of kidney function

The race-free CKD-EPI (2021) equation was used to calculate eGFR from serum creatinine measures.^17^ CKD was defined as an eGFR ≤60 mL/min/1.73 m^2^.

### Exposures and additional variables

Through manual chart review, we assessed how many patients had creatine kinase (CK, U/L) level between their first and second scan. We also quantified the number of patients who received abdominal radiotherapy, reported lymphedema, had statin or steroid exposure, or a grade 3 or 4 irAE throughout the course of the study.

### Statistical methods

Summary statistics for the body composition parameters (mean, 95% confidence interval [CI]) were computed at baseline and follow-up for the non–ICI and ICI groups. Data were examined for normality and paired *t-*tests were used to examine the change in body composition parameters (SMI, PMD) between baseline and follow-up. A “difference in change” was defined by establishing the change in body composition measurements from baseline to follow-up and comparing this change between the ICI and non–ICI groups. Independent *t* tests were used to determine whether there were differences in the mean change from baseline to follow-up between the non–ICI and the ICI groups for each measurement. For any significant declines within the ICI group, we assessed whether these changes also occurred in patients without a grade 3 or 4 irAE or progressive disease throughout the study. After compiling the mean change in SMI and PMD in each group, we performed a post-hoc power calculation to detect a significant difference in change between groups for our analyses.

To control for differences in age and sex, multivariable linear regression models evaluating difference in change of SMI and PMD (between non–ICI and ICI groups) were used. We additionally adjusted for the participant’s baseline SMI and PMD values. For any significant results, we tested lymphedema and statin exposure as a co-variates. Bonferroni correction was used to correct for multiple comparisons as we had 2 primary outcomes (adjusted difference in change in SMI and PMD). Therefore, the *P*-values for the fully adjusted difference in change of SMI and PMD between the ICI and the non–ICI groups were multiplied by 2, and results were considered significant if these adjusted *P*-values were less than 0.05. To determine if baseline eGFR modifies the relationship between change in SMI and PMD among groups, baseline eGFR and baseline eGFR x ICI exposure interaction term were added to the sex and age-adjusted models. Statistical analyses were performed using STATA version 15.1.

## Results

### Patient characteristics

Baseline characteristics of the 41 patients not treated with ICIs and 36 patients treated with ICIs are shown in Table 1. The non–ICI group was older as compared to the ICI group (67.0 ± 12.9 vs. 58.9 ± 14.9 years, respectively) and more likely male (78% vs. 50%). The majority of patients in the ICI group (27 [75%]) were treated with PD-1 inhibitors and 9 (25%) were treated with a combination of PD-1 and CTLA-4 inhibitors. Eighteen (50%) patients in the ICI group were on statins compared with 16 (39%) in the non–ICI group. Only 1 patient in each group received abdominal radiotherapy throughout the course of the study. Four patients who were not treated with ICIs and 11 patients treated with ICI’s had CK measurements between their first and second scans. The median CK (range) of values of 107 U/L (24 to 220). No patients had CK levels over the upper limit of normal. Of the patients who had several levels of CK during this timeframe, the largest change in values was an increase from 155 to 220 U/L with subsequent decline to 183 U/L. No myositis was reported on CT reports.

**Table 1.** Baseline characteristics.

	ICI group (*N* = 36)	Non–ICI group (*N* = 41)	*P*-value
**Age, years** ^a^	58.9 ± 14.9	67.0 ± 12.9	0.01
**Male**	18 (50.0%)	32 (78.0%)	0.01
**Melanoma stage**	III	II	
**Interval between baseline and follow-up scans (days)** ^a^	384.8 ± 62.2	359.6 ± 46.8	0.05
**Comorbidities**			
**Diabetes**	7 (19.4%)	11 (26.8%)	0.45
**Smoking within 1 year of baseline scan**	2 (5.6%)	4 (9.8%)	0.76
**Hypertension**	10 (27.8%)	16 (39.0%)	0.30
**Measurements**			
**Estimated glomerular filtration rate**	85 (19)	74 (19)	0.01
**Body mass index (BMI), kg/m^2^**	30.96 (7.53) (*N* = 7)	29.44 (8.34) (*N* = 15)	0.69
**Medications at baseline**			
**Proton pump inhibitor**	6 (18.8%)	5 (12.2%)	
**SGLTi**	0 (0.0%)	4 (9.8%)	
**GLP-1 agonist**	0	1 (0.2%)	
**Statins**	18 (50%)	16 (39%)	
**Initial ICI therapy**			
**Combination ICI**	9 (25.0%)		
**PD-(L)1 inhibitor monotherapy**	27 (75%)		
**Cytotoxic T-lymphocyte–associated protein 4 inhibitor monotherapy**	0		
**Unknown**	0		
**Duration of therapy (days)** ^a^	375.61 ± 134.60		

a Expressed in mean ± standard deviation.

ICI = immune checkpoint inhibitor.

At baseline, the mean eGFR in the ICI group was 85 ± 19 mL/min/1.73 m^2^. Mean eGFR in the non–ICI group was 74 ± 19 mL/min/1.73 m^2^. Four patients in the ICI group and 10 in the non–ICI group had CKD at baseline.

### Skeletal muscle index

**Figure 2.**
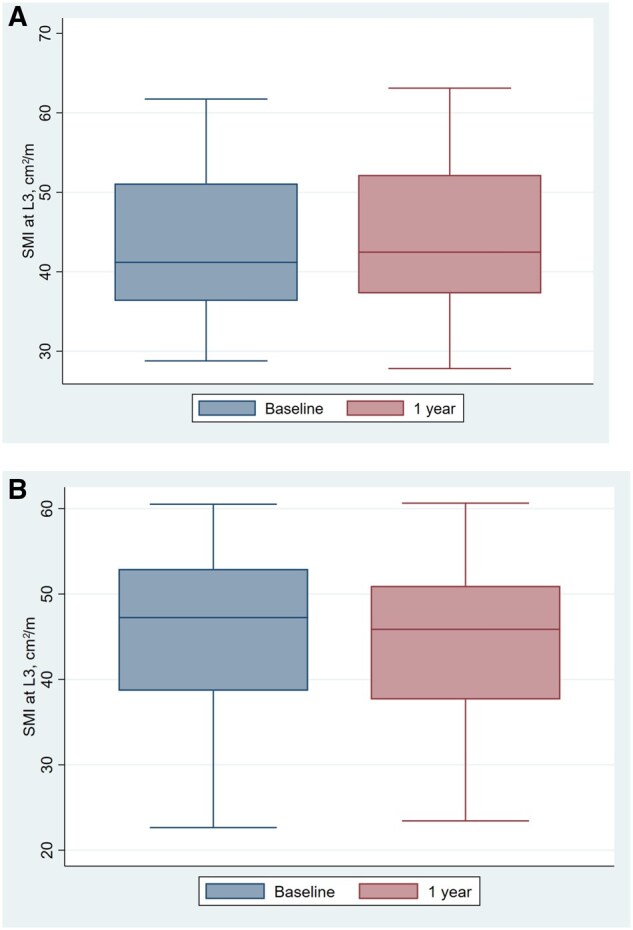
Skeletal muscle index (SMI, cm/m^2^) at baseline and 1 year in: (A) immune checkpoint inhibitor (ICI) group; (B) non–ICI Group.

**Figure 3.**
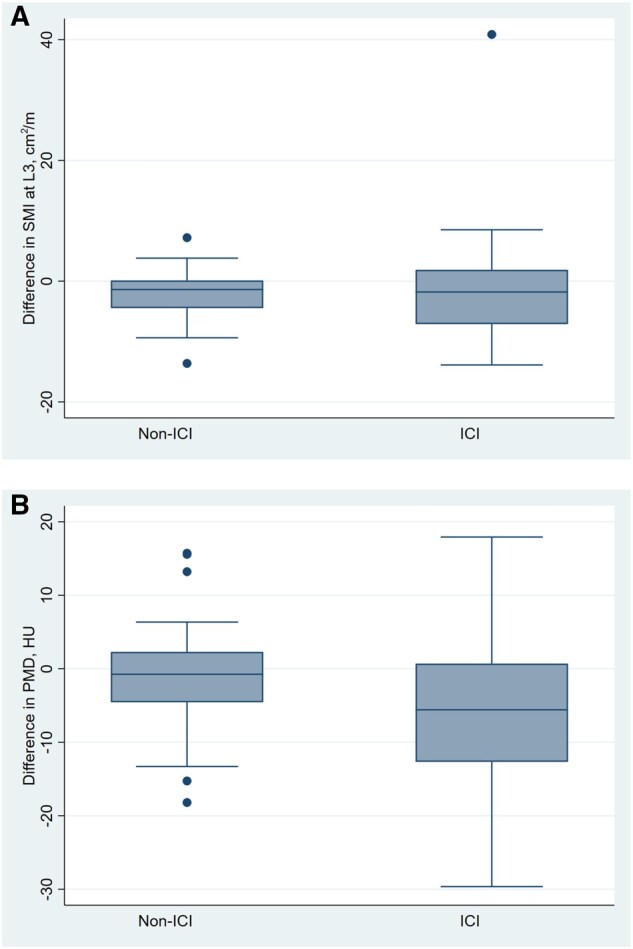
Skeletal muscle index (SMI) and psoas muscle density (PMD) difference in change from baseline to follow-up in: (A) immune checkpoint inhibitor (ICI) group; (B) non–ICI group.

The baseline and follow-up SMI measurements in the ICI and non–ICI groups are shown in Table 2 and Figure 2. The difference in SMI between baseline and 1 year is shown in Figure 3A. In unadjusted analyses, non–ICI group had significant changes in SMI at 1 year (−2.07 [−3.37-−0.78]; *P* = 0.002). The ICI group did not have a significant change in SMI (−0.46 [−4.63-3.72]; *P* = 0.82). The difference in change between non–ICI and ICI groups were not significant (*P* = 0.38). Based on our post-hoc power calculation utilizing the mean change and standard deviation of change in each group, we had 99.2% power to detect a significant difference in change in SMI between groups (alpha = 0.05).

**Table 2.** Mean body composition measurements with 95% confidence intervals (CI) at baseline and follow-up, stratified by ICI usage.

	Baseline			Follow-up	Change	*P*-value
**SMI, cm^2^/m (CSA, cm^2^ at L3 divided by patient height in m)**						
**ICI**		75.16 (67.41-82.92)		74.71 (67.24-82.18)	−0.46 (−4.63-3.72)	0.82
**Non–ICI**		79.91 (74.41-85.42)		77.83 (74.41-85.42)	−2.07 (−3.37-−0.78)	**0.002**
**Difference in change** ^a^					1.62 (−2.06-5.30)	0.38
**Psoas muscle density, HU (HU at L3)**						
**ICI**		37.33 (33.83-40.84)		31.40 (26.92-35.89)	−5.93 (−9.58-−2.28)	**0.002**
**Non–ICI**		35.72 (32.18-39.26)		34.54 (31.13-37.96)	−1.18 (−3.51-1.15)	0.31
**Difference in change**					−4.75 (−8.89-−0.61)	**0.03**
**eGFR**						
**ICI (*n* = 33)**		84.42 (78.05-90.80)	84.06 (76.93-91.19)		−0.36 (−3.92-3.19)	0.84
**Non-ICI (*n* = 21)**		72.86 (63.07-82.64)	71.86 (64.38-79.34)		−1 (−5.46-3.46)	0.64
**Difference in change**					0.64 (−4.93-6.20)	0.82

a Non–ICI—ICI.

eGFR = estimated glomerular filtration rate, mL/min/1.73m^2^; ICI, immune checkpoint inhibitor; SMI = skeletal muscle index.

Baseline and follow up values display mean body composition measurements stratified by immune checkpoint inhibitor (ICI) use. Values are presented with 95% confidence intervals (CI). Bold p values are significant findings (*p* < 0.01).

In multivariate models adjusted for age, sex, and baseline value, the difference in change between non–ICI and ICI groups remained non–significant (*P* = 1.00). Male gender (β [95% CI] = 2.63 [−0.05-6.00]; *P* = 0.13) and age (β [95% CI] = 0.03 [−0.05-0.11]; *P* = 0.43) were not associated with change in SMI.

### Psoas muscle density

**Figure 4.**
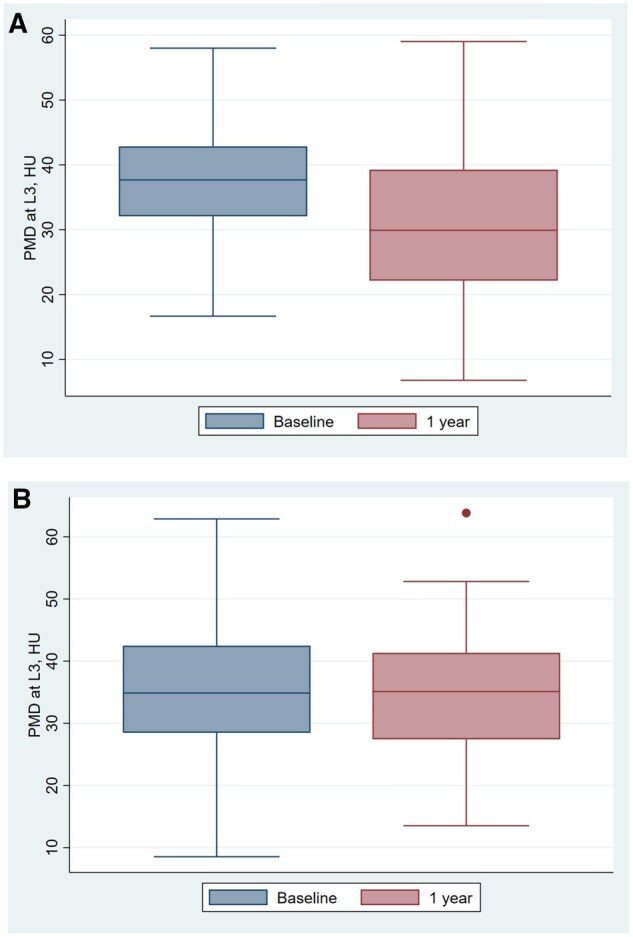
Psoas muscle density (PMD) at the 3rd lumbar vertebra (L3) at baseline and 1 year in (A) immune checkpoint inhibitor (ICI) group; (B) non–ICI group.

The baseline and follow-up psoas muscle density (PMD) measurements in ICI and non–ICI groups are shown in Table 2 and Figure 4. The difference in PMD between baseline and 1 year is shown in Figure 3B. In unadjusted analyses, the non–ICI group did not have a significant change in PMD over 1 year (−1.18 [−3.51-1.15]; *P* = 0.31). The ICI group had a significant change in PMD (−5.93 [−9.58-−2.28]; *P* = 0.002). After excluding patients who experienced a grade 3 or 4 irAE, a significant decline in mean (SD) PMD was still noted (37.5 HU [10.7] to 31.6 HU [13.7] [*P* = 0.007]).The difference in change was significantly different between groups (−4.75 [−8.89-−0.61]; *P* = 0.002), with the ICI group exhibiting a significantly greater decline in PMD, as compared with the non–ICI group. Adjusting for statin exposure did not significantly change our results (mean [95% CI] difference between groups = −5.50 HU [−9.95-−1.04], *P* = 0.02). The interaction term of statin exposure × ICI exposure was not significant, and thus stratified analysis was not performed (β = −8.3, *P* = 0.07). Similarly, adjusting the presence of lymphedema did not change our outcomes (adjusted difference in change −5.55 HU [−10.36-−0.74], *P*-value = 0.02) nor did disease progression (adjusted difference in change −5.49 HU [−9.97-−1.01], *P*-value = 0.02). Subgroup analyses removing patients with progressive disease still demonstrated significant declines in patients without progressive disease (mean [SD] HU dropped from 37.8 [9.4] to 32.4 [13.3], *P* = 0.02).

Significant differences in IMAT and IMAT ratio were not observed in either group (Table S2—see online supplementary material). Based on our power calculation utilizing the mean change and standard deviation of change in each group, we had 100% power to detect a significant difference in change in PMD between groups (alpha = 0.05).

Using the non–sex-specific definition of low PMD, the number of patients in the non–ICI group with low PMD increased slightly from 23 of 41 (56%) at baseline to 25 of 41 (61%) at follow up. The number of patients in the ICI group with low PMD increased from 19 of 36 (53%) to 26 of 36 (72%) at follow up. Similar results were seen using the sex-specific definition of low PMD. At baseline, 22 of 41 (54%) of patients in the non–ICI group had low PMD and 24 of 41 (59%) had low PMD at follow up. Among patients in the ICI group, 13 of 36 (36%) met criteria for low muscle density at baseline and 21 of 36 (58%) had low PMD at follow-up.

In multivariate models adjusted for age, sex, and baseline value (Table 3), the difference in PMD remained significantly different between groups (−6.84 [−10.94-−2.73]; *P* = 0.001). Higher age (β [95% CI] = −0.26 [−0.43-−0.10]; *P* = 0.002) was significantly associated with change in PMD. Male gender was not associated with change in PMD (β [95% CI] = 2.17 [−1.96-6.30]; *P* = 0.30).

**Table 3.** Adjusted difference in change in skeletal muscle index and psoas muscle density with 95% confidence interval (CI) between ICI and non–ICI groups.

	Difference adjusted for age and sex (95% CI)	*P*-value	Difference adjusted for age, sex and baseline value (95% CI)	*P*-valuea	eGFR x ICI exposure interaction term
**Skeletal muscle index (CSA cm^2^ at L3 divided by patient height in m)**	0.08 (−2.23-2.40)	0.94	0.18 (−2.17-2.54)	1.00	0.49
**Psoas muscle density**	−6.00 (−10.5-−1.51)	0.01	−6.84 (−10.94-2.73)	0.001	0.38

* Bonferroni adjusted *P*-value.

eGFR = estimated glomerular filtration rate; ICI, immune checkpoint inhibitor.

### Associations with baseline eGFR

Baseline eGFR was not significantly associated with change in SMI (β [95% CI] = 0.003 [−0.07-0.08]; *P* = 0.95) in multivariate models adjusting for age, sex, and baseline value. Baseline eGFR × ICI exposure interaction term was not significant (*P* = 0.49) in subsequent models. Baseline eGFR was not significantly associated with change in PMD (β [95% CI] = 0.04 [−0.10-0.18]; *P* = 0.58) in multivariate models adjusting for age, sex, and baseline value. Baseline eGFR × ICI exposure interaction term was not significant (*P* = 0.38) in subsequent models.

## Discussion

This study observed significantly higher changes in PMD in patients treated with ICIs as compared to patients not on ICI therapy. There was no significant difference in change in skeletal muscle mass as measured by SMI in both patients treated with and without ICIs. Pre–treatment kidney function did not modify the association between ICI use and change in SMI or PMD in our study.

Low muscle density is associated with deteriorations in muscle strength,^18–20^ poor physical function,^18^^,^^20–22^ hospitalizations,^23^ fracture,^24^ prolonged hospitalization,^25^^,^^26^ and greater disability.^18^ Further, higher PMD on abdominal CT scans has also been associated with overall survival in patients with metastatic renal cell carcinoma,^27^ pancreatic cancer,^28^ esophageal,^29^ and breast cancer.^30^ Therefore, preservation of muscle in patients undergoing cancer therapy is an important concern for patient quality of life and longevity.

Specifically, CT-derived measures of PMD have demonstrated clinically relevant associations with functional and health outcomes. In a cohort of 174 elderly patients, lower CT measures of PMD were associated with impaired balance.^21^ In a HealthABC study of 1527 patients ages 70-79, 1 HU higher PMD at baseline was associated with a higher physical function score 4 years later (*P* < 0.01).^22^ The score was composed of time to complete 5 chair stands, timed standing balance, timed usual pace 6 min walk, and timed narrow 6 min walk. Similarly, a 5 HU increase in PMD was associated with shorter hospital and ICU stay in patients following liver transplant (*R*^2^ = 0.22 and 0.14, respectively).^25^ Therefore, the observed a −5.93 (95% CI: −9.58-−2.28) HU change in PMD in the patients treated with ICIs in our study is consistent with clinically meaningful change.

Further, using the 25th percentile cutoff derived from a cohort of 151 adult patients ages ≥45 at Ohio State University admitted with blunt trauma; the number of patients treated with ICIs who had low PMD increased from 13 at baseline to 21 on follow-up. In the derivation cohort, these cutoffs were associated with higher 90-day mortality (RR 5.95, *P* < 0.008) length of stay ≥7 days (RR 1.63, *P* = 0.048), complication risk (RR 2.30, *P* = 0.002), and dependent status at discharge (2.14, *P* = 0.015). These findings further highlight the clinical relevance of PMD decline during ICI therapy that may continue in subsequent years with continued exposure.

Importantly, our study did not include patients treated with ≥5 mg prednisone-equivalent for 3 months or more and the observed differences in declines in PMD were present even in patients without documented irAE of grade 3 or 4. Therefore, the proposed mechanism of our observed declines in PMD in patients on ICI therapy as compared to patients not on ICI therapy is likely independent of clinically significant myositis or higher steroid exposure. Notably, the non–ICI group had stage II melanoma, whereas the ICI group had stage III disease; however, results remained significant after excluding patients with cancer progression during the study period.

Both clinical and preclinical models note loss of muscle strength prior to loss of muscle mass.^31^^,^^32^ Compared to patients not treated with ICI’s, we observed a significant decline in PMD but not SMI in patients treated with ICI’s without a significant change in IMAT. IMAT measures the visible storage of lipids in adipocytes located between the muscle fibers and between muscle groups^33^ and is separately segmented from muscle area. By contrast, PMD captures the density of the muscle itself and thereby can reflect fatty deposition within the muscle cells. Therefore, our observed decline in PMD without declines in SMI may be lipid infiltration,^34^ subclinical inflammation, or muscle fiber injury, which can occur without substantial loss of muscle cross-sectional area or increase in IMAT.

Pre–treatment kidney function did not impact the relationship between ICI exposure and change in SMI or PMD. CKD is associated with sarcopenia across the spectrum of CKD stages^6^ and sarcopenia is independently associated with mortality in the general population regardless of CKD status.^35^ Notably, our cohort did not have advanced CKD at baseline limiting this assessment and only 14 patients had an eGFR ≤60 mL/min/1.73 m^2^. However, eGFR measurements often inaccurately assess true kidney function as compared to gold standard measured GFR^36^ such that patients with low muscle mass at baseline may have an overestimate of their serum creatinine based eGFR. This is of particular importance as at baseline, 38 (92%) non–ICI and 27 (75%) ICI patients met criteria for sarcopenia based on age and sex cutoffs for SMI^1^; therefore, the true GFR in our cohort is likely worse than the reported serum creatinine eGFR. Considering kidney function and frailty are important exclusion criteria for various cancer therapies and clinical trials, additional studies are needed to determine whether these factors lead to adverse outcomes in patients started on newer cancer therapies as conditions such as sarcopenia may actually improve with effective cancer treatment.

Our study had several limitations. While this is the largest study to date exploring muscle changes on CT in patients treated with ICIs, the sample size is modest. Nonetheless, we had sufficient power to detect significant difference in change between groups. In both cohorts, some patients were scanned on different CT scanners at baseline and follow-up, introducing uncertainty about how this may influence PMD estimates derived from scan HUs. Further, the scans are uncalibrated and our analyses compared measurements obtained from different scanners, sites, and protocols. However, the proportion of patients in the ICI and non–ICI groups who were scanned on different machines was similar, suggesting that any scanner-related variability would likely impact both groups equally. The assessment of muscle quantity and quality was restricted to intra–abdominal muscles, which may not capture whole-body muscle changes or histological changes detected only via muscle biopsy. Furthermore, functional outcomes such as physical performance or quality of life were not assessed, limiting the clinical interpretation of changes in muscle metrics. Residual confounding due to unmeasured factors such as nutritional status, or inflammatory burden may influence results. The non–ICI group, by indication, had less advanced cancer at baseline. While the observed findings may be confounded by cancer stage at baseline, we specifically chose a cohort of patients with stage II melanoma not treated with ICIs compared to stage III patients treated with ICIs, instead of patients with stage IV melanoma treated with ICIs because we felt that there was less likely to be systemic differences from the cancer itself between stage II and III, both of which do not have extra-nodal cancer metastases. Of note, previous work has shown some stage II melanoma patients (B/C) have a worse prognosis than stage III A/B patients.^37^ Lastly, while the strict inclusion criteria used in this study minimized potential confounders, it limits the generalizability of our results and prevents us from assessing associations with advanced CKD. However, given that the impact of ICIs on SMI and PMD in humans was previously unknown, we employed strict inclusion criteria to minimize confounding in exchange for decreased generalizability. Future work could increase generalizability by expanding to other cancer types and stages, broader range of baseline eGFR, various age groups, and those exposed to glucocorticoids.

This study also has numerous strengths and addresses a novel and understudied area: the impact of ICI therapy on skeletal muscle health, particularly in the context of kidney function. It leverages objective and quantifiable imaging-based measurements (SMI and PMD) derived from clinically acquired CT or PET-CT scans, enhancing real-world applicability. The inclusion of a comparison group of patients with cancer not receiving ICIs allows for contextualization of changes in muscle characteristics attributable to treatment. The use of multivariable modeling to adjust for baseline differences between groups strengthens the validity of the findings. Lastly, the focus on the intersection of immunotherapy, muscle wasting, and kidney function may have important implications for vulnerable patient populations with CKD.

In conclusion, we observed significantly larger declines in muscle density in patients being treated with ICI as compared to those not treated with ICIs. Clinicians should be cautious when patients are starting ICI therapy as observed declines in muscle density may have important implications for their overall physical function and longevity during and after the course of treatment. While treatment of their cancer is of utmost importance, accelerated declines in muscle quality may have important long-term consequences for their survivorship journey. Further research is needed to confirm ICI-associated muscle loss, better understand its mechanism, and develop intervention strategies.

## Supplementary Material

oyag029_Supplementary_Data

## Data Availability

The data may be available upon reasonable request for research purposes to researchers by submitting a proposal in writing to Dr Carrie Ye.
