# Supplementary material for: Association of immune checkpoint inhibitors with muscle mass and density in patients with melanoma
Source: Oncologist. 2026 Feb 12;31(3):oyag029. doi: 10.1093/oncolo/oyag029 (PMC12959323; doi:10.1093/oncolo/oyag029)
Supplement: oyag029_Supplementary_Data [file oyag029_supplementary_data.docx]

**Supplemental Material.**

**Table S1**. Summary of the different manufacturers and image parameters of the scans used for the opportunistic computed tomography (CT) analysis.

|  | | GE Healthcare | Philips | Canon Medical Systems | Siemens Healthineers |
| --- | --- | --- | --- | --- | --- |
| Model | Discovery CT750 HD, Discovery MI,  Revolution HD | | Gemini TF TOF 16 | Aquilion, Aquilion One | Biograph 40,  Biograph 128,  Biograph 128 Edge,  SOMATOM Definition,  SOMATOM Definition AS+,  SOMATOM Definition Edge,  SOMATOM Definition Flash |
| Voltage (kVp) | 120, 140 | | 100, 120, | 120 | 100, 120, 140 |
| Slice Thickness (mm) | 0.625, 1.25, 2.5 | | 3 | 1, 2 | 0.6, 1.5, 2 |
| Reconstruction kernel | Standard | | B, D | FC04, FC05, FC08, FC18 | B19f, B35f, B40f, I30f, I40f, I41f |

**Table S2.**

| Mean intermuscular adipose tissue measurements with 95% confidence intervals (CI) at baseline and follow-up, stratified by ICI usage. | | | | | |
| --- | --- | --- | --- | --- | --- |
|  | Baseline | | Follow-up | Change | p-value |
| **IMAT, cm^2^ (mean CSA at L3 and L4)** | | | | | |
| ICI | | 10.17 (6.58 – 13.75) | 10.55 (6.58 – 14.51) | 0.38 (-1.28 – 0.52) | 0.40 |
| Non-ICI | | 11.78 (9.19 – 14.36) | 12.84 (10.02 – 15.67) | 1.07 (-2.24 – 0.10) | 0.07 |
| Difference in change | |  | | -0.69 (-2.18 – 0.80) | 0.36 |
| **IMAT ratio (mean IMAT CSA at L3 and L4/ mean muscle CSA at L3 and L4)** | | | | | |
| ICI | | 0.09 (0.06 – 0.12) | 0.10 (0.06 – 0.14) | -0.01 (-0.02 – 0.004) | 0.15 |
| Non-ICI | | 0.11 (0.07 – 0.14) | 0.11 (0.08 – 0.15) | -0.008 (-0.02 – 0.002) | 0.12 |
| Difference in change | |  | | 0.002 (-0.02 – 0.02) | 0.84 |

IMAT= intermuscular adipose tissue
